# Induction of Hair Growth by Insulin-Like Growth Factor-1 in 1,763 MHz Radiofrequency-Irradiated Hair Follicle Cells

**DOI:** 10.1371/journal.pone.0028474

**Published:** 2011-12-02

**Authors:** Sun-Young Yoon, Kyu-Tae Kim, Seong Jin Jo, A-Ri Cho, Soon-Ik Jeon, Hyung-Do Choi, Kyu Han Kim, Gun-Sik Park, Jeong-Ki Pack, Oh Sang Kwon, Woong-Yang Park

**Affiliations:** 1 Department of Dermatology, College of Medicine, Seoul National University, Seoul, Korea; 2 Department of Biomedical Sciences, College of Medicine, Seoul National University, Seoul, Korea; 3 Department of Physics and Astronomy, College of Natural Sciences, Seoul National University, Seoul, Korea; 4 Radio Technology Research Department, Communications & Broadcasting Covergence, Electronics and Telecommunications Research Institute, Daejeon, Korea; 5 Electromagnetic Research Center, Chungnam National University, Daejeon, Korea; City of Hope National Medical Center and Beckman Research Institute, United States of America

## Abstract

Radiofrequency (RF) radiation does not transfer high energy to break the covalent bonds of macromolecules, but these low energy stimuli might be sufficient to induce molecular responses in a specific manner. We monitored the effect of 1,763 MHz RF radiation on cultured human dermal papilla cells (hDPCs) by evaluating changes in the expression of cytokines related to hair growth. The expression of insulin-like growth factor-1 (IGF-1) mRNA in hDPCs was significantly induced upon RF radiation at the specific absorption rate of 10 W/kg, which resulted in increased expression of B-cell chronic lymphocytic leukemia/lymphoma 2 (BCL-2) and cyclin D1 (CCND1) proteins and increased phosphorylation of MAPK1 protein. Exposure to 10 W/kg RF radiation 1 h per day for 7 days significantly enhanced hair shaft elongation in *ex vivo* hair organ cultures. In RF-exposed follicular matrix keratinocytes in the hair bulb, the expression of Ki-67 was increased, while the signal for terminal deoxynucleotidyl transferase dUTP nick end labeling was reduced. From these results, we suggest that 1,763 MHz RF exposure stimulates hair growth *in vitro* through the induction of IGF-1 in hDPCs.

## Introduction

The biological effects of radiofrequency (RF) radiation in the frequency range of 10 kHz to 300 GHz emitted from wireless devices such as cellular phones has been studied for more than 40 years [1]. Such an RF radiation does not induce DNA damage because RF radiation does not transfer high enough energy to break the covalent bonds of macromolecules. Nonetheless, the possibility of bio-interaction between RF radiation and human cells and macromolecules remains to be clarified in a tissue-specific and stage-specific manner [2].

A large number of investigations about RF exposure have been conducted in various *in vitro* and *in vivo* models. Numerous molecular approaches demonstrated no biological effects on cellular signaling, mRNA or protein expression [3], [4], [5], yet the thermal effects of RF radiation may be applied to biomedical fields to treat human diseases [6], [7]. There are many investigations indicating controversial or inconclusive results of cellular changes after RF radiation with various frequencies [1].

The diverse opinions about the cellular changes mediated by RF radiation may be determined by experimental conditions such as the model system/cell type, exposure time, frequency, and radiation dose [8]. We have previously reported characteristics of biological effects of RF exposure on auditory hair cells [9]. Auditory hair cells could easily be exposed to mobile phone frequency and 1,763 MHz RF exposure, but this exposure did not induce cellular responses, including cell cycle distribution, DNA damage, stress response, or gene expression changes at 20 W/kg specific absorption rate (SAR) in HEI-OCI auditory hair cells.

RF ablation (RFA) provides a controlled heating modality on microscopic tissue targets based on RF electrical current flow (≤500 kHz) [10]. Furthermore, a variety of models have been proposed to explain the mechanism of wound healing by electrical stimulation. Previous studies using high-voltage pulsed galvanic stimulation showed that the electrical stimulus induced cell migration and wound repair through increased protein and DNA synthesis [11]. Maddin et al. demonstrated that a pulsed electrostatic field had positive biological effects on hair re-growth but the biophysical mechanism was not clear [12].

The dermal papilla is a discrete population of specialized fibroblasts and plays a pivotal role in hair formation, growth, and cell cycling [13]. To explore the effect of RF radiation in human hair follicle (HF) cells, we irradiated human dermal papilla cells to code division multiple access-type 1,763 MHz RF radiation and monitored alterations at molecular and cellular levels. In *ex-vivo* organ culture of hair follicles, we measured hair shaft elongation after RF exposure. Our results suggest that RF exposure could stimulate human hair growth *in vitro*.

## Results

**Figure 1:**
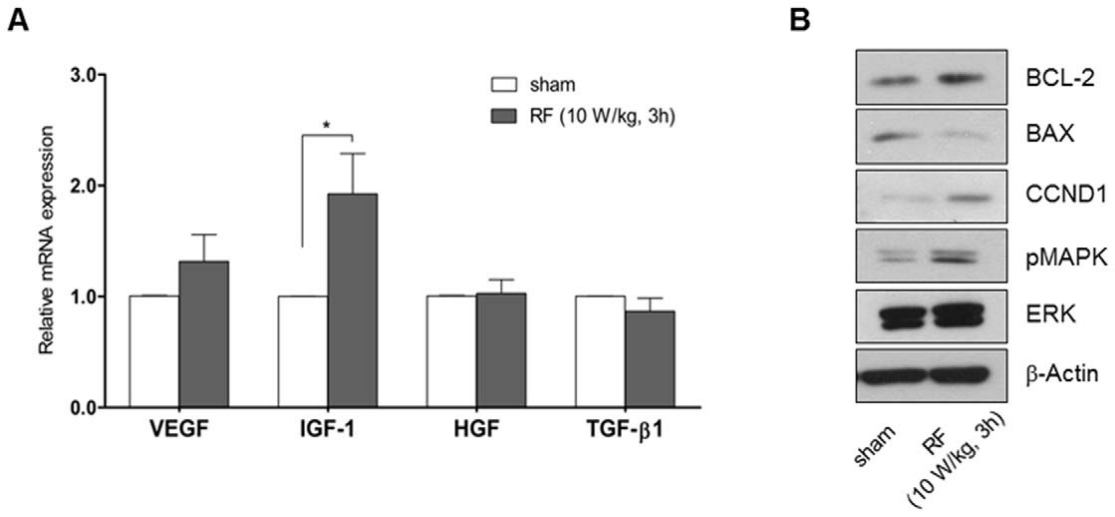
Growth factor expression in RF-exposed hDPCs. All samples were isolated from human HF tissues from different individuals. Total RNA from DPCs was prepared after 1,763 MHz RF radiation at 10 W/kg SAR for 3 h. mRNA expression levels of VEGF, IGF-1, HGF, and TGF-β1 were measured by real-time PCR (A). Results are expressed as mean ± standard error (SE). Cells were lysed immediately after 1,763 MHz RF radiation at 10 W/kg SAR for 3 h and analyzed by Western blotting against BCL-2, BAX, pMAPK, MAPK and β-Actin (B).

Growth factors play a critical role in the regulation of morphogenesis, cell proliferation and growth of human hairs. We screened the expression levels of key growth factors such as vascular endothelial growth factor A (VEGF), insulin-like growth factor 1 (IGF-1), hepatocyte growth factor (HGF) and transforming growth factor beta 1 (TGF-β1) by quantitative reverse transcription-polymerase chain reaction (RT-PCR) in RF-exposed hDPCs. After exposure to 1,763 MHz RF radiation at an SAR of 10 W/kg for 3 h, mRNA expression levels of IGF-1 and VEGF, both of which can stimulate hair growth, increased (Fig. 1A). The mRNA expression level of TGF-β1, an inhibitor of hair growth, did not change compared with sham-exposed cells. We found no change in HGF mRNA expression.

RF exposure induced the expression of IGF-1 mRNA, which may lead to altered protein expression downstream of IGF-1. The mitogen-activated protein kinase (MAPK) signaling pathway can be activated in response to IGF-1 to promote cell survival [14]. We performed Western blotting to analyze the expressions of proteins related to the MAPK pathway after RF radiation. We found that RF exposure at an SAR of 10 W/kg for 3 h increased the phosphorylation of MAPK1 in cultured hDPCs (Fig. 1B). In addition, RF exposure increased B-cell chronic lymphocytic leukemia/lymphoma 2 (BCL-2) and cyclin D1 (CCND1) expression and decreased BCL-2-associated X protein (BAX) levels in cultured hDPCs as well (Fig. 1B).

**Figure 2:**
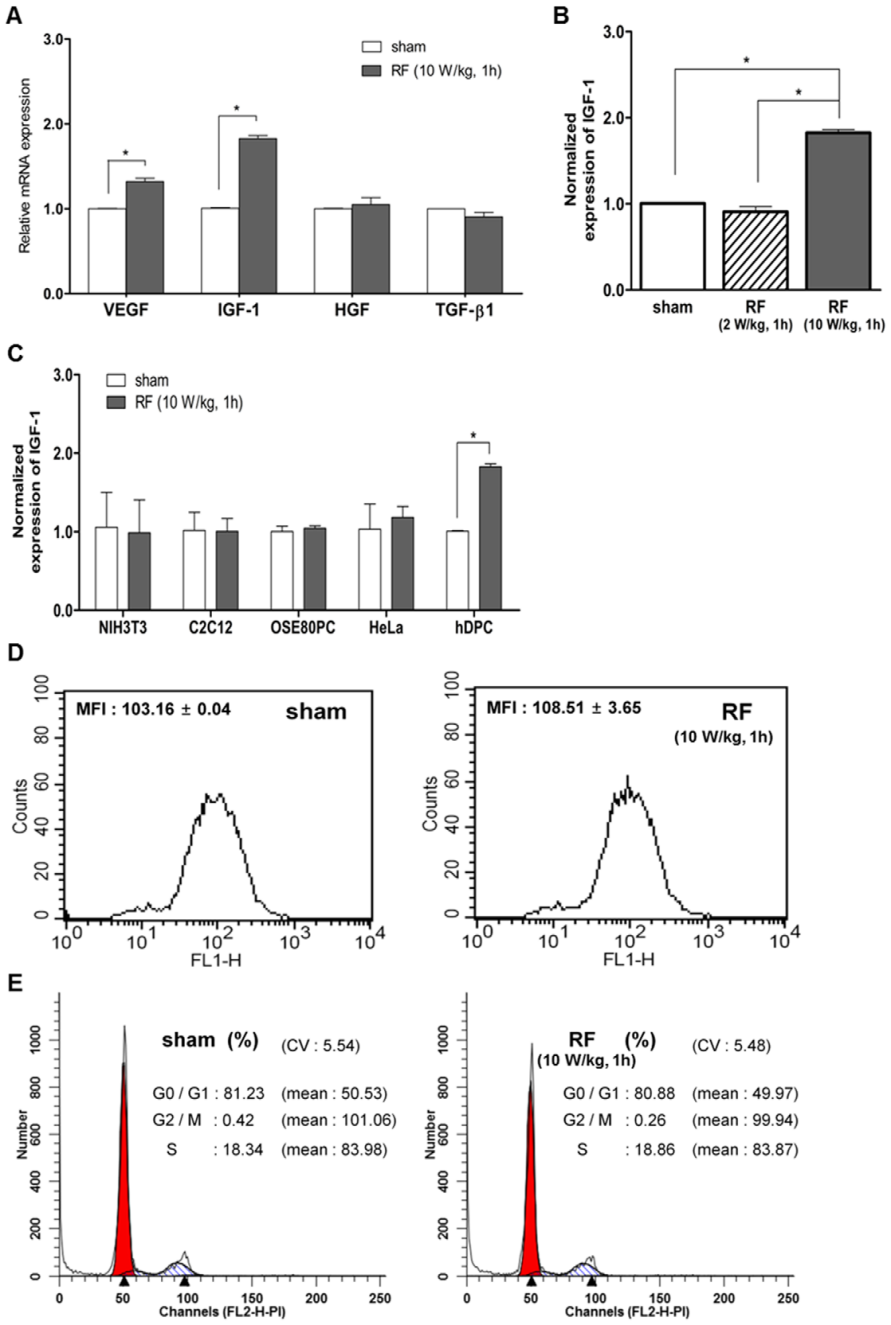
IGF-1 expression in various conditions. hDPCs were exposed to RF radiation at an SAR of 10 W/kg for 1 h and mRNA expression levels of VEGF, IGF-1, HGF, and TGF-β1 were measured by real-time PCR (A). We also exposed hDPCs to 1,763 MHz RF radiation at 2 W/kg for 1 h to measure the induction of IGF-1 mRNA (B). Under the same conditions of RF exposure, we irradiated NIH3T3, C2C12, OSE-80PC, and HeLa cells and measured IGF-1 mRNA (C). The cell cycle distributions of sham and RF-exposed hDPCs were also measured by PI staining and FACS analysis (D). Analysis of cell cycle distribution showed no significant difference between two samples in three repetitive experiments. Results are expressed as mean ± SE. *P<0.05 versus non-irradiated control.

We examined whether IGF-1 was also induced in other exposure conditions. First, we shortened the exposure duration to 1 h and found significant induction of IGF-1 and VEGF mRNAs (Fig. 2A). On the other hand, RF radiation at a lower SAR of 2 W/kg for 1 h did not induce IGF-1 mRNA expression (Fig. 2B). Moreover, the effect of RF radiation on the induction of growth factors was specific to hDPCs because we did not detect any change in IGF-1 mRNA expression in four different cell lines, including NIH3T3 mouse fibroblasts, C2C12 mouse myoblasts, OSE-80PC normal human ovarian epithelial cells, and HeLa human cervical cancer cells (Fig. 2C).

The biological effects of RF radiation are still controversial and some studies have demonstrated its hazardous effect *in vivo* as well as *in vitro*. Oxidative stress is a result of the imbalance between reactive oxygen species (ROS) and antioxidants in the body [15], which can induce DNA damage, apoptosis, and necrosis of the cells. To determine if oxidative stress occurred by RF radiation in hDPCs, we measured ROS levels by incubating hDPCs with DCF-DA (2′,7′-dichloro-fluorescein diacetate; 10 µM) and using oxidation-based fluorescence and fluorescence-activated cell sorting (FACS) analysis. We analyzed ROS production in hDPCs immediately after exposure to 1,763 MHz RF at an SAR of 10 W/kg for 1 h, but we did not detect any significant change in the ROS value compared to sham-exposed hDPCs (Fig. 2D). The cell cycle distribution of RF-exposed hDPCs was similar to that of sham-exposed hDPCs as well (Fig. 2E).

**Figure 3:**
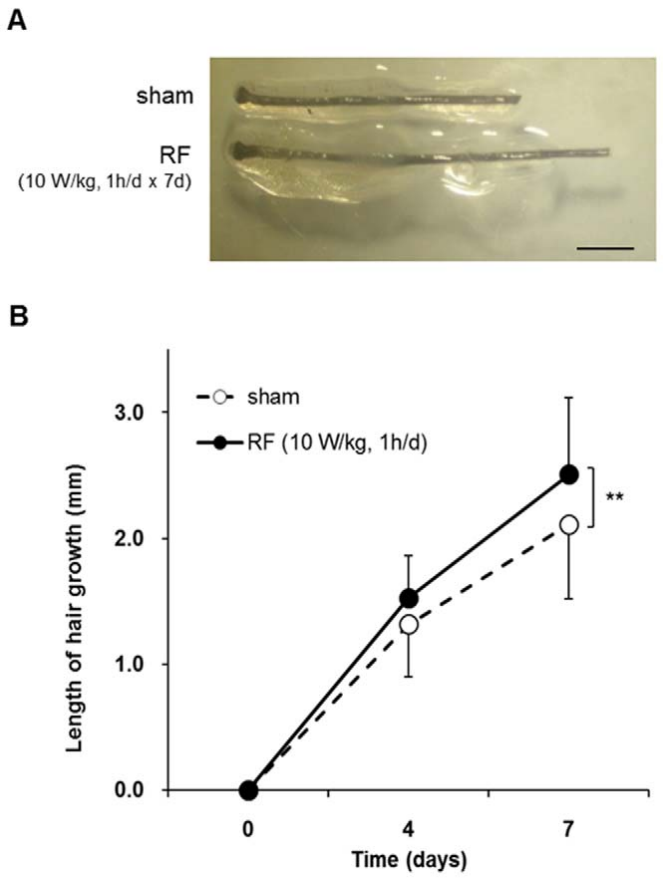
Elongation of hair shaft induced by RF exposure in *ex vivo* hair organ culture. Dissected human scalp HFs were exposed to RF radiation for 1 h everyday with 10 W/kg SAR over a period of 7 days. Hair shafts were cultured *ex vivo* and elongation measured for the indicated periods (A). A total of 90 anagen HFs from three different volunteers (30 follicles per subject) were cultured (B). **P<0.01 versus non-irradiated control. Results are expressed as mean ± SE. Scale bar represents 1 mm.

Next we examined the effect of RF radiation at organ level using *ex vivo* cultured whole human scalp HFs. Human HFs were exposed to 1,763 MHz RF at 10 W/kg for 1 h per day for 7 days. The length of 90 HFs per group was measured after 7 days of culture and we found that hair shaft was significantly elongated in RF-exposed group compared to that of sham exposure. RF exposure stimulated the growth of organ-cultured HFs significantly (Fig. 3A, 3B, p<0.01).

**Figure 4:**
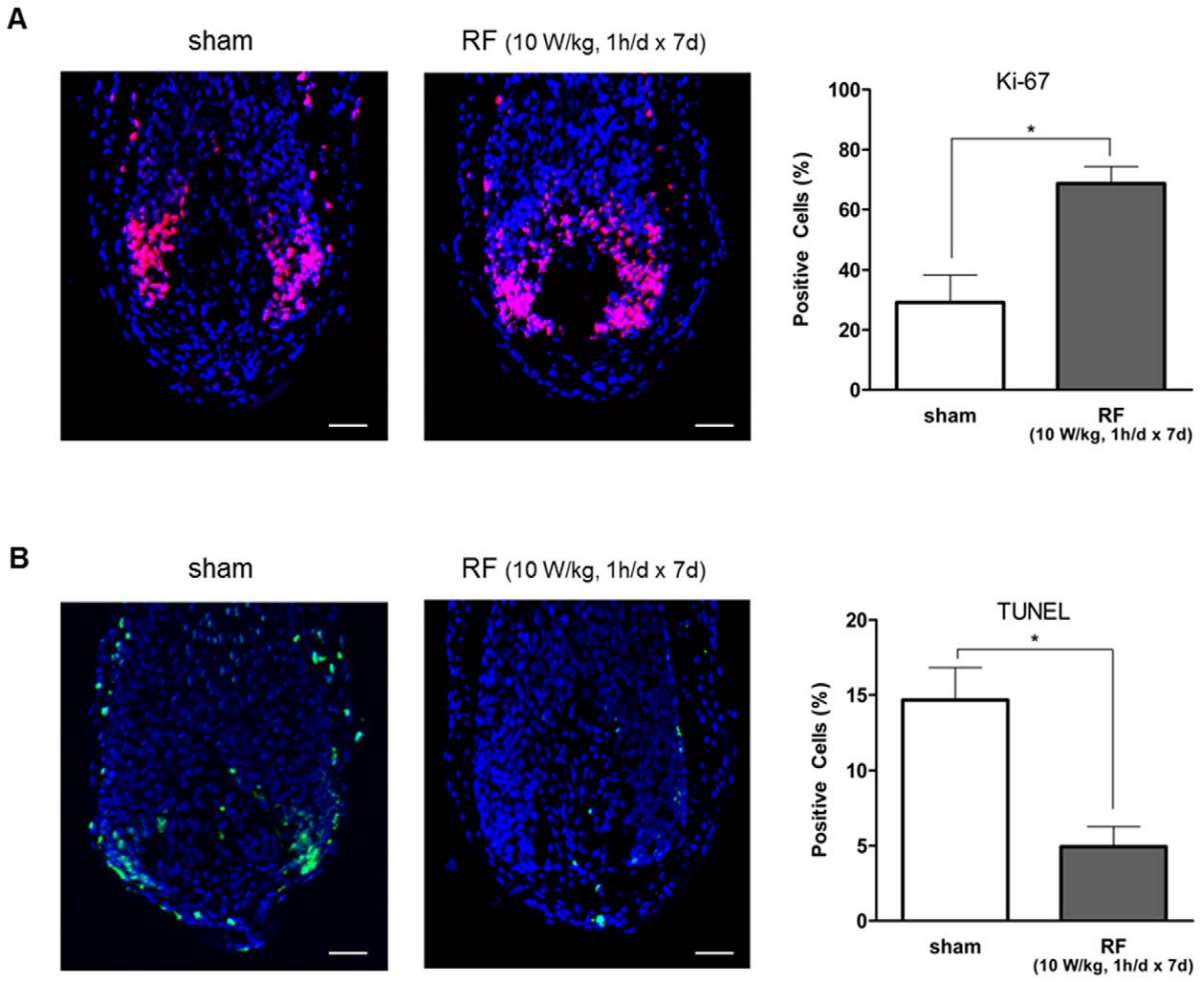
Expression of molecular markers for cellular proliferation and apoptosis in RF-exposed hair matrix keratinocytes. Immunofluorescence staining of Ki-67 (A) and TUNEL (B) was applied after 1,763 MHz RF irradiation on human HFs for 1 h every day at an SAR of 10 W/kg for 7 days. HF sections derived from three different individuals were analyzed for proliferation (Ki-67+, red fluorescence, A) and apoptosis (TUNEL+, green fluorescence, B) in the keratinocyte of the hair bulb. Nuclei were counterstained with DAPI (blue fluorescence). For quantitative analyses, the number of TUNEL+ or Ki-67+ cells was counted and normalized to the number of DAPI-stained cells. *P<0.05 versus non-irradiated control. Results are expressed as mean ± SE. Scale bar represents 50 µm.

We determined whether RF radiation could affect the cell growth or cell death in organ culture HFs. We performed immunofluorescence staining for Ki-67 and terminal deoxynucleotidyl transferase dUTP nick end labeling (TUNEL) after human HFs were exposed to 1,763 MHz RF irradiation for 1 h every day at an SAR of 10 W/kg over a 7-day period. HF sections derived from three different individuals were analyzed for proliferation (Ki-67+, red fluorescence, Fig. 4A) and apoptosis (TUNEL+, green fluorescence, Fig. 4B) in follicular matrix keratinocytes in the hair bulb. Nuclei were counterstained with DAPI (4′,6-diamidino-2-phenylindole; blue fluorescence). For quantitative analyses, the number of TUNEL+ or Ki-67+ cells was counted and normalized to the number of DAPI-stained cells. Compared with sham-exposed cells, RF exposure significantly increased the number of Ki-67+ keratinocytes (p<0.05) and decreased the number of TUNEL+ keratinocytes (p<0.05). These results show that RF exposure induced proliferation and suppressed apoptosis in hair matrix keratinocytes.

## Discussion

Various studies have been conducted to determine the biological effects of RF radiation, but it has not yet been determined if RF radiation poses a potential hazard [16]. The biological effect of RF radiation remains controversial and the results from different studies may vary due to different experimental conditions and model systems [8]. In an effort to find cell type-specific responses to RF radiation in our previous study, we tested the effect of 1,763 MHz RF radiation on Jurkat human T cells [8] and HEI-OC1 mouse auditory hair cells using microarray analysis [9]. However, we could not find significant alteration in gene expression and cell signaling. In this study, we examined the effect of RF exposure on primary cultured human HFs and hDPCs to find the increased hair growth of ex vivo cultured HFs through the expression of growth factors such as IGF-1.

Hair growth is observable in the anagen stage before the regression phase (catagen) and the resting phase (telogen) of the hair follicle cycle [17], [18]. Growth factors are polypeptides that are involved in the regulation of hair morphogenesis, hair cell proliferation, and hair growth [19], [20]. Some reports have provided evidence that growth factors such as VEGF, IGF-1, and HGF have hair growth stimulatory activity, while TGF-β1 has inhibitory effects on hair growth [21], [22]. It has been reported that IGF-1, VEGF, fibroblast growth factor 5, and fibroblast growth factor 7 induce proliferation of cells in the matrix, dermal papilla, and dermal papillary vascular system and increase the amount of extracellular matrix in dermal papilla [23]. These factors also maintain follicles in the anagen phase [23], [24], whereas TGF-β1 evokes apoptosis of matrix cells and shifts the follicles from anagen to catagen [24], [25]. IGF-1 is recognized as a potent stimulator of HF growth and an important physiologic regulator of the hair growth cycle [23], [26], [27]. We screened the mRNA expression levels of growth factors and found that IGF-1 mRNA expression levels increased after RF radiation at 10 W/kg for 1 h. On the other hand, other growth factors such as VEGF, HGF and TGF-β1 were not changed, which suggests that the expression of IGF-1 was rather specific to RF radiation. The increased expression of IGF-1 was validated by Western blot against BCL-2, BAX, and pMAPK1, all of which seem to be targets of IGF-1 [28], [29].

RF exposure activates IGF-1 mRNA expression to promote cell survival of hDPCs by increasing the ratio of BCL-2/BAX. CCND1 plays an important role in promoting G1-to-S phase progression [30]. The signals downstream of MAPK related to mitogenesis contain the phosphorylation of transcription factors including JUN, ETS1, and ELK1, and synthesis of cell cycle regulatory proteins such as the cyclin D family. RF exposure could up-regulate the phosphorylation of MAPK1 and subsequently increase the expression of CCND1 as shown in Fig. 1. However, the overall cell cycle progression was not changed in RF-exposed cells, as shown in Fig. 2E. The increased levels of BCL-2 and CCND1 and the phosphorylation of MAPK1 may not be sufficient to induce the proliferation of hDPCs. The expression of BCL-2 and CCND1 and the phosphorylation of MAPK1 are important for cell survival by inhibiting apoptosis [31]. Cell cycle progression requires additional changes in gatekeeper genes such as p53 and Rb for G1/S transition [32]. We could not detect changes in cell cycle distribution in RF-irradiated hDPCs as other type of cells.

RF exposure does not induce DNA strand breaks, chromosome aberrations, sister chromatid exchanges, DNA repair synthesis, phenotypic mutation, or transformation such as cancer-like changes [33]. Exposure to RF radiation exerts no detectable effects on cell cycle distribution, cellular migration, or invasion at average SAR values of 2 or 10 W/kg [16]. In contrast, another study noted that RF radiation induced basal cell proliferation and mild skin changes in rat skin cells [34]. A diverse range of opinions exist with regard to whether RF exposure induces cellular change, and the results of these studies depend on the cell type, exposure time, frequency, or radiation dose used [8]. We used higher SAR levels than previous reports, but we did not detect any evidence of oxidative stress.

In this study, we demonstrated that RF exposure stimulated hair growth in an *ex vivo* system and an *in vitro* model using hDPCs. Our future studies will be focused on investigating the effect of RF radiation using various frequencies and doses to better understand the biological responses. In addition, we need to explore human hair growth on other types of HFs. Interestingly, 1,763 MHz RF radiation at a higher SAR, for example 60 W/kg, hardly showed linear responses in hDPCs and outer root sheath cells, which are keratinocytes of human scalp HFs (data not shown). From these results, we propose that RF radiation at a specific dose and exposure time could be used to treat human hair disorders such as androgenetic alopecia.

## Materials and Methods

### Ethics statement

The study was approved by the Institutional Review Board at the Seoul National University Hospital, and all subjects gave written informed consent. All experimental procedures using human materials have been conducted according to the principles described in the Declaration of Helsinki.

### RF exposure apparatus

A customized exposure unit was used for *in vitro* and *ex vivo* experiments as previously published [35]. Briefly, the designated RF signal at 1,763 MHz was generated from a Code Domain Multiple Access (CDMA) source and delivered to a monopole antenna in rectangular cavity-type TE_102_ mode chamber after passing through amplifiers and an attenuator. Considering where the electric field was maximized in the chamber, the feeding antenna was at the λ_g_/8 position of the top plate of the cavity and the 100 mm-culture dish was put at the λ_g_/4 position of the bottom. The uniform distribution of electric field and SAR in culture dish was simulated by the Finite Difference Time Domain method (XFDTD, Remcom, Inc., State College, PA, USA). The output power of SAR and exposure time schedule was controlled by Graphic User Interface (GUI).

For exclusion of the thermally induced effects on samples, a cooling device was equipped to control the temperature rise of cell culture media by water circulation through the bottom of the chamber. The temperature inside the culture dish was monitored with a fluoroptic thermal probe 790 (Luxtron Corp., Santa Clara, CA, USA) with thermal resolution of 0.1°C and we periodically checked that temperature was maintained at 37.0 ± 0.2°C, including under RF radiation.

### Exposure conditions

Cells or human HFs were prepared for RF radiation exposure in 100-mm culture dishes containing 18 mL of culture media indicated below. The relevant volume of 18 mL was characterized by numerical simulations, using the FDTD to set the height of culture medium in a 100-mm culture dish to 4 mm for 1,763 MHz frequency specific resonance in the TE_102_ mode chamber. Samples were exposed at SAR values of 2 and 10 W/kg.

For the sham exposure group, all conditions were uniformly applied to the samples except that they received mock RF radiation exposure. Cells in the RF irradiated chamber and sham exposure chamber were placed in an incubator (US/NU-5500G, NuAire Inc., Plymouth, MN, USA) to automatically maintain CO_2_ level and humidity inside the chamber.

### Isolation of human HFs and dermal papilla

Skin biopsy specimens were obtained from the occipital scalp region of healthy volunteers who had not received any medication for at least 1 month. HFs were isolated under a stereo dissecting microscope, and dermal papilla was microdissected from individually isolated HFs as previously described [36], [37]. HFs considered to be in the anagen stage morphologically were used in this study.

### Cell culture

The methods used for isolating and culturing hDPCs have been described previously [37], [38]. hDPCs were cultured in Dulbecco's Modified Eagle's Medium (DMEM; Welgene, Daegu, Korea) containing 10% fetal bovine serum (FBS; Welgene), 10 ng/mL basic fibroblast growth factor (R&D Systems, Minneapolis, MN, USA), and 1X antibiotic antimycotic solution (Gibco BRL, Gaithersburg, MD, USA) containing penicillin and streptomycin. Cells were incubated at 37°C, in a 5% CO_2_ incubator. To study the effect of RF exposure, hDPCs were serum-starved for 24 h and then cells were exposed to RF for the indicated time.

Four other cell lines including NIH3T3 [39], C2C12 [40], OSE-80PC [41], and HeLa [42] cells were each cultured in different cell culture media. NIH3T3 and HeLa cells were cultured in DMEM supplemented with 10% FBS and 1% antimycotic solution. For OSE-80PC cells, RPMI 1640 medium (WelGENE, Daegu, Korea) was used with 10% FBS and 1% antimycotic solution. To culture C2C12 cells in an undifferentiated state, 20% FBS and 1% antimycotic solution were included in the DMEM.

### HF organ culture and immunofluorescence staining

Human scalp HFs were isolated and cultured as described previously [37], [43]. Each dissected HF was cut into a piece of approximately 3–3.5 mm in length from the bottom of dermal papilla and cultured in Williams E medium (Gibco BRL) with 10 ng/mL hydrocortisone, 10 µg/mL insulin, 2 mM L-glutamine, and 1X antibiotic antimycotic solution (Gibco BRL) containing penicillin and streptomycin. Follicles were maintained at 37°C in a 5% CO_2_ atmosphere. HFs were irradiated for 1 h every day at an SAR of 10 W/kg over a 7-day period. In all experiments, the tissue culture medium was changed every 2 days. HF elongation was measured directly after 7 days of culture using an Olympus stereo microscope with an eyepiece containing a graticule. A total of 90 HFs from three different volunteers (30 follicles per volunteer) were analyzed in each growth condition.

To evaluate proliferating cells, we performed immunofluorescence staining with an antibody to Ki-67 (DAKO, Carpinteria, CA, USA). Cultured HFs were embedded in OCT (Leica Biosystems, Richmond, IL, USA) and frozen in liquid nitrogen. Cryosections of 5-µm thickness were postfixed in 4% paraformaldehyde for 20 min at room temperature. After several washes in phosphate buffered saline (pH 7.4), the sections were blocked with blocking solution (DakoCytomation, Ely, UK) for 30 min and then incubated with the primary mouse monoclonal antibody against Ki-67 at 4°C for 18 h. After washing in phosphate buffered saline, the sections were incubated with Alexa Fluor 594-labeled goat anti-mouse immunoglobulin G (IgG; Invitrogen Japan, Tokyo, Japan) for 1 h at room temperature. The nuclei were counterstained with DAPI.

To measure apoptotic cells we performed TUNEL labeling (*In Situ* cell death detection kit, fluorescein, Roche Diagnostics, Mannheim, Germany). Cryostat sections were fixed in 4% paraformaldehyde and incubated in permeabilization solution at 4°C for 2 min. The sections were then labeled with a digoxigenin-deoxyUTP in the presence of terminal deoxynucleotidyl transferase. TUNEL+ cells were visualized with an anti-digoxigenin FITC-conjugated antibody. All sections were examined immediately after the concluding TUNEL labeling and photographed with a Zeiss LSM 510 META confocal laser scanning microscope (Zeiss, Jena, Germany). Immunofluorescence images were acquired by using the Zeiss LSM 510 META confocal microscopy software and confocal parameters for the pinhole, optical slide, detector gain, amplifier offset, amplifier gain, contrast, and brightness were kept constant. For quantitative analyses, the number of TUNEL+ or Ki-67+ cells was counted and normalized to the number of DAPI-stained cells by using 3-dimensional software (Imaris Version 6.1, Bitplane AG, Switzerland).

### Quantitative real time-PCR

Total RNA was isolated from hDPCs using RNA iso Plus (Takara Bio Inc., Otsu, Shiga, Japan) and treated with DNase I (Roche Pharamceuticals, Welwyn Garden City, UK) to remove the genomic DNA. We used 1 µg of total RNA for the cDNA synthesis reaction, which was performed using a First Strand cDNA Synthesis Kit (Fermentas, St Leon-Rot, Germany) according to the manufacturer's instructions. To quantitatively estimate the mRNA expression of VEGF, HGF, IGF-1, and TGF-β1, PCR was performed on a 7500 Real-time PCR System (Applied Biosystems, Foster City, CA, USA) using the SYBR® Premix Ex Taq™ (Takara Bio Inc.) according to the manufacturer's instructions.

The following primers for human genes were used: GAPDH (forward, 5′-CAA TGA CCC CTT CAT TGA CC-3′; reverse, 5′-AAA TGA GCC CCA GCC TTC T-3′), VEGF (forward, 5′-CCA TGA ACT TTC TGC TGT CTT-3′; reverse, 5′-TCG ATC GTT CTG TAT CAG TCT-3′), IGF-1 (forward, 5′- CTC TTC TAC CTG GCG CTG TG -3′; reverse, 5′- CAT ACC CTG TGG GCT TGT TG-3′), HGF (forward, 5′- AGA AAT GCA GCC AGC ATC AT-3′; reverse, 5′-CAC ATG GTCC TGA TCC AAT C-3′), TGF-β1 (forward,5′-GCC CTG GAC ACC AAC TAT TG-3′; reverse,5′ GTC CAG GCT CCA AAT GTA GG-3′). For mouse cell lines, the following primers were used: GAPDH (forward, 5′-GGC ATG GAC TGT GGT CAT GAG-3′; reverse, 5′-TGC ACC ACC AAC TGC TTA GC-3′), IGF-1 (forward, 5′-ACC GAG GGG CTT TTA CTT CA-3′; reverse 5′-CTC CTC AGA TCA CAG CTC CG-3′). The PCR reaction was carried out according to the manufacturer's instructions. All samples were run in triplicate with SYBR Green and independently repeated three times.

### Western blot analysis

Protein from hDPCs was extracted using RIPA lysis buffer (Millipore, Billerica, MA, USA). Soluble extracts were prepared by centrifugation at 15,000 rpm for 15 min at 4°C. Protein (20 µg per lane) was separated using 10% sodium dodecyl sulfate-polyacrylamide gel electrophoresis and transferred to a polyvinylidene fluoride membrane (Amersham, Buckinghamshire, UK). The blotted membrane was incubated with primary antibodies at 4°C overnight. The following antibodies were used: anti-total MAPK1, anti-phosphorylated MAPK1, anti-Bcl-2, anti-Bax, anti-cyclinD1 (Cell Signaling Technology, Beverly, MA, USA), and anti-ß-actin (Santa Cruz Biotechnology, Santa Cruz, CA, USA). The membrane was probed with anti-mouse IgG-horseradish peroxidase conjugates or anti-rabbit IgG-horseradish peroxidase conjugates (Santa Cruz Biotechnology) for 1 h at room temperature. The antibody–antigen complexes were detected using the enhanced chemiluminescence system (Amersham Pharmacia Biotech, Little Chalfont, UK). The results were analyzed using a Bio-Rad GS-700 imaging densitometer (Hercules, CA, USA).

### Measurement of ROS

To check for the presence of oxidative stress, the production of ROS was measured using the oxidation-sensitive probe DCF-DA (Invitrogen, Carlsbad, CA, USA). DCF-DA (10 µM) was used as the ROS detection reagent and FACS cytometry performed according to the manufacturer's instructions. After RF at an SAR of 10 W/kg for 1 h, we assessed ROS generation in the hDPCs. All samples were analyzed independently three times.

### Analysis of cell cycle distribution

Cell cycle analysis was performed with PI (Sigma-Aldrich, Steinheim, Germany) staining. After RF exposure to the hDPCs at 10 W/kg SAR for 1 h, trypsinized cell pellets were suspended in serum-free cell culture media. The cells were then fixed with 70% ethanol and incubated at 4°C for 1 h. Cells were stained with propidium iodide (50 µg/mL) supplemented with RNaseA (Sigma-Aldrich, Steinheim, Germany) at 4°C for 30 min before analysis by flow cytometry. All samples were independently analyzed and repeated three times.

### Statistical analysis

Statistical significance was determined using a student's t-test. P-values are two-tailed and significance was accepted at P<0.05. In the case of organ culture, a paired student's t-test was used for statistical analysis.
